# Cost-effectiveness analysis of newborn pulse oximetry screening to detect critical congenital heart disease in Colombia

**DOI:** 10.1186/s12962-019-0179-2

**Published:** 2019-06-24

**Authors:** Dario Londoño Trujillo, Nestor Fernando Sandoval Reyes, Alejandra Taborda Restrepo, Cindy Lorena Chamorro Velasquez, Maria Teresa Dominguez Torres, Sandra Vanessa Romero Ducuara, Gloria Amparo Troncoso Moreno, Hernan Camilo Aranguren Bello, Alejandra Fonseca Cuevas, Pablo Andres Bermudez Hernandez, Pablo Sandoval Trujillo, Rodolfo Jose Dennis

**Affiliations:** 1 Public Health Division, Fundacion Santa Fe de Bogota, Carrera 7 B # 123–90, 5 Piso, Bogotá, Colombia; 2 Institute of Congenital Heart Disease, Fundacion Cardioinfantil-Institute of Cardiology, Bogotá, Colombia; 3 Research Department, Fundacion Cardioinfantil-Institute of Cardiology, Bogotá, Colombia; 4 Neonatal Unit, Pediatrics Department, Fundacion Cardioinfantil-Institute of Cardiology, Bogotá, Colombia; 5 School of Medicine, Universidad de los Andes, Bogotá, Colombia

**Keywords:** Cost-effectiveness analysis, Congenital heart defects, Diagnosis, Pulse oximetry, Indirect expenditures

## Abstract

**Background:**

In many countries, economic assessments of the routine use of pulse oximetry in the detection of Critical Congenital Heart Disease (CCHD) at birth has not yet been carried out. CCHDs necessarily require medical intervention within the first months of life. This assessment is a priority in low and medium resource countries. The purpose of this study was to assess the cost-effectiveness (CE) relation of pulse oximetry in the detection of cases of CCHD in Colombia.

**Methods:**

A full economic assessment of the cost-effectiveness type was conducted from the perspective of society. A decision tree was constructed to establish a comparison between newborn physical examination plus pulse oximetry, versus physical examination alone, in the diagnosis of CCHDs. The sensitivity and specificity of pulse oximetry were estimated from a systematic review of the literature; to assess resource use, micro-costing analyses and surveys were conducted. The time horizon of the economic evaluation was the first week after birth and until the first year of life. The incremental cost-effectiveness ratio (ICER) was determined and, to control for uncertainty, deterministic and probabilistic sensitivity analysis were made, including the adoption of different scenarios of budgetary impact. All costs are expressed in US dollars from 2017, using the average exchange rate for 2017 [$2,951.15 COP for 1 dollar].

**Results:**

The costs of pulse oximetry screening plus physical examination were $102; $7 higher than physical examination alone. The effectiveness of pulse oximetry plus the physical examination was 0.93; that is, 0.07 more than the physical examination on its own. The ICER was $100 for pulse oximetry screening; that is, if one wishes to increase 1% the probability of a correct CCHD diagnosis, this amount would have to be invested. A willingness to pay of $26.292 USD (direct medical cost) per probability of a correct CCHD diagnosis was assumed.

**Conclusions:**

At current rates and from the perspective of society, newborn pulse oximetry screening at 24 h in addition to physical examination, and considering a time horizon of 1 week, is a cost-effective strategy in the early diagnosis of CCHDs in Colombia.

*Trial registration* “retrospectively registered”.

## Background

Critical congenital heart diseases (CCHD) make up a group of structural defects of the heart that are present from the prenatal period and represent more than a third of all congenital heart cardiopathies [1, 2]; at world level, their incidence ranges from 1 in 15,000 to 1 in 26,000 live births and their prevalence is 147.4 per 100,000 live births [3]. Amongst the main CCHDs we find *Pulmonary Atresia, Tetralogy of Fallot, Tricuspid Atresia, Truncus Arteriosus, Hypoplastic Left Heart Syndrome, Total Anomalus Pulmonary Venous Return and the Transposition of Great Vessels.* These diseases generate an important morbidity and mortality burden from the first month of the infant’s life, and hence it is necessary to perform surgical and/or early interventional treatment [2, 4].

The early detection of these cardiopathies can help to significantly modify the clinical course of patients with CCHD. This detection may take place in different ways before birth, as in the case of prenatal ultrasound and anatomic ultrasound testing. However, prenatal detection of these cases is still underused in many countries. Almost 30% of newborns affected are diagnosed late [5], which means an untimely medical-surgical intervention, with a high morbidity and mortality rate [6].

After birth, CCHDs may be identified by physical examination within the first 24 h and through other diagnostic tests like EKG or chest X-ray; however, these tests lack the necessary sensitivity to detect most cases [6]. For this reason, it is necessary to consider other early detection techniques such as pulse oximetry, which is a highly sensitive, well-established, non-invasive test for the objective quantification of hypoxemia, which may be suitable for the routine screening of CCHD [7, 8]. Use of this screening method for early detection of congenital heart defects is based on the rationale that clinically undetectable hypoxemia is present, to some degree, in most potentially life-threatening cases. Pulse oximetry has been previously assessed as a screening method for congenital heart defects in newborns [8]. The primary benefit of newborn screening for CCHD with pulse oximetry is timely identification before hospital discharge, thereby minimizing the morbidity and mortality associated with delayed diagnosis [9–11].

From the standpoint of technology assessment, pulse oximetry has shown to be cost-effective in countries like the United States, the United Kingdom and China [12]. In studies published conducted in these countries, mainly from the perspective of the health system and with a time horizon of less than a year, comparing pulse oximetry with the clinical general examination ended with the correct diagnosis of CCHD or the number of deaths avoided [13–15].

In countries like Colombia, the universal use of pulse oximetry 24 h after birth, in addition to standardized physical examination of the newborn as a strategy for screening congenital heart disease is recommended [16, 17]. However, in spite of the recommendations and availability of the technology, clinical experts on the subject state that this clinical practice has not been widely accepted on a regular basis in Colombia: a local study on the subject showed that only 25% of the physicians in the survey know and apply neonatal screening in a correct way [18].

The purpose of this study was to assess the cost-effectiveness of pulse oximetry plus physical examination in the correct and timely detection of CCHDs, when compared with physical examination alone, and to estimate the likely budget impact of its gradual implementation in clinical practice, as a new national policy.

## Methods

### Economic assessment

A cost-effectiveness study from the societal perspective was proposed. This study included direct and indirect costs associated with the outcomes, which are covered by the general social security system (SGSSS) and families, and compared the use of pulse oximetry screening in addition to general physical examination with the general physical examination alone. Health outcomes were measured as correct diagnosis and survival. The target population of the study was a hypothetical cohort of non-premature newborns, up to the first 24 h after birth.

Prognosis in CCHD is directly dependent on a timely diagnosis; therefore, the time horizon for the economic evaluation was defined according to two related outcomes: firstly, (a) the probability of a correctly diagnosed CCHD at 1 week of age; and secondly, (b) survival at 12 months. Both assumptions were considered based on the natural history of the disease (according to the literature and the consensus of clinical experts in the management of CCHD). In view of the time horizon of less than a year, it was not necessary to apply discount rates.

**Fig. 1:**
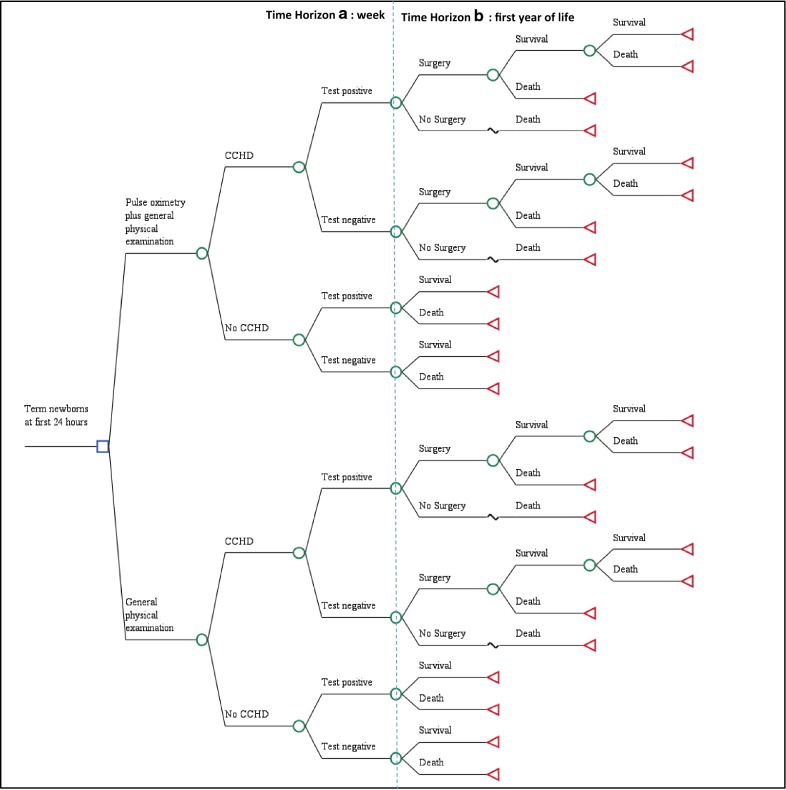
Decision tree

With the purpose of estimating costs and the potential benefits, a decision tree was proposed which reflects the possible outcomes for the term newborn diagnosed by alternatives compared within the time horizon defined: (a) after one week (cases correctly diagnosed) and (b) the first year of life (overlife) (Fig. 1).

In this model it was assumed that the general examination corresponds to the examination conducted by the general, not specialized physician. In the case of a positive diagnosis for CCHD (with any alternative), confirmation tests were included according to the assistance algorithm depending on the altitude above sea level. The altitude above sea level at which oximetry (SatO2) is measured influences the SatO2 cut-off point chosen to rule-out disease (as altitude increases, atmospheric pressure and SatO2 decrease in normal subjects as well) [19]. Colombia has an altitude range varying from zero to 6000 m above sea level (MASL), and its more densely populated geographical areas are located between 1000 and 2700 MASL. Thus, the design of a screening algorithm with different SatO2 cutoff points (allowing for different MASL) was necessary. During the first week, indirect costs relevant to the families are not included. Lastly, it was considered that if newborns had a CCHD diagnosis and were not medically intervened within the first year, mortality rates would reach 100%.

Expected costs and outcomes of each strategy were estimated in TreeAge Pro ^®^ 2017.

#### Effectiveness

The choice of health outcomes (correct diagnosis and survival) were validated with experts in the diagnosis and treatment of CCHDs. In order to estimate event probabilities in the model, four systematic literature reviews (SLR) were conducted: sensitivity and specificity of both tests: pulse oximetry associated with general physical examination, and general physical examination alone [20]; the likely prevalence of CCHD; and CCHD mortality estimates with and without-delayed surgical treatment. The search for SLRs on the prevalence of CCHDs was performed in Pubmed, Embase, Ovid, Scopus, LILACS and TRIPDatabase. We included longitudinal, prospective, retrospective, cross-sectional, cohort, and case studies published between 2011 and 2016, published in English, Spanish or French. The SLR on the sensitivity and specificity of both tests (pulse oximetry associated with the general physical examination and the general physical examination alone) was conducted in Pubmed, Science Direct, Ovid and EBSCO; in this review we included systematic reviews, meta-analysis, case and control studies and cohort studies published between 2002 and 2016, regardless of the language. For the SLR on CCHDs mortality, a search was made in Scopus and Pubmed including retrospective studies in English, French and Spanish from 2011 to 2017, and for the SLR on mortality post-surgical intervention, the search was made in Pubmed, Sciencedirect, LILACS, Ebsco- Host, Cochrane, Scopus, including cohort and case and control studies in English and Spanish, between 2012 and 2017 (Table 1).

**Table 1:** Effectiveness parameters used in the model

Effectiveness	Base (average)	Minimum	Maximum	Source
Prevalence of CCHD	0.014	0.006	0.032	SRL
Sensitivity of general examination	0.5878	0.115	0.892	SRL
Specificity of general examination	0.8631	0.4	0.99	SRL
Sensitivity of pulse oximetry	0.8869	0.8276	0.955	SRL
Specificity of pulse oximetry	0.9325	0.665	0.998	SRL
CCHD mortality	0.143	0.026	0.795	SRL
Mortality post-intervention	0.0917	0.018	0.1702	SRL
General mortality under 1 year in Colombia	0.017	0.01	0.02	DANE [21]

*CCHD* critical congenital heart disease, *DANE* administrative department of national statistics

Given that the probability of death as a result of late diagnosis was not found in the literature, it was estimated following the methodology suggested by Grigore et al. [22, 23] to obtain event probabilities from clinical experts. For this, clinical experts (15 pediatric cardiologists) were given two scenarios and asked to give their estimate on the proportion of patients who would die in each one of them: (A) the patient has a CCHD, diagnosis is confirmed, but for some reason s/he does not undergo surgery; and (B) the patient has a CCHD, the diagnosis is not considered and s/he does not undergo surgery (Table 2).

**Table 2:** Effectiveness parameters not available in the literature

Effectiveness parameters	Base (average)	Minimum	Maximum
Probability of death with no surgery but diagnosis was confirmed	0.39	0.08	0.94
Probability of death with no surgery and diagnosis not considered (false negative)	0.69	0.17	0.93

#### Costs

To estimate direct costs, a micro-costing analysis was made by reviewing the clinical records of 73 CCHD patients from the databasaes of a hospital specialized in the management of CCHDs in the city of Bogota. To estimate indirect costs, a survey was applied to 20 caregivers of patients with CCHDs. This survey asked caregivers about the out-of-pocket expenses incurred by family and any days of leave from work related with patient care. Indirect costs were established by means of the human capital approach, with average daily income calculated based on the distribution of reported income by all caregivers surveyed. Medical direct costs were valued at market prices, using as reference standard fees from Colombia´s Social Security manual. Generally, contracts between insurers and providers of health services is based on this national tariff manual (called ISS 2001); prices in this manual are adjusted in the negotiation, and 35% represents the most common current mark-up used for most economic evaluations in Colombia. All costs are given in USD, using the average exchange rate for 2017 [$2,951.15 pesos for 1 dollar] [24] (Table 3).

**Table 3:** Cost parameters used in the model

Costs	Base (average)	Minimum	Maximum	Source
Pulse oximetry + general examination	$ 60	$ 25	$ 117	Own calculations ISS 2001
General examination	$ 19	$ 9	$ 35	ISS 2001
Confirmatory tests	$ 530	$ 425	$ 1055	ISS 2001
Average cost hospital events for CCHD	$ 25,835	$ 16,904	$ 42,419	Cost estimation through clinical records, ISS 2001 valuation
Ambulatory cost of CCHD	$ 457	$ 449	$ 533	Construction with experts, ISS 2001 valuation
Indirect costs of CCHD with no surgery	$ 775	$ 42	$ 1000	Construction by authors through surveys of caregivers
Indirect costs of CCHD with surgery	$ 1466	$ 333	$ 3802	
Out-of-pocket expenses with no surgery	$ 1083	$ 28	$ 3106	
Out-of-pocket expenses with surgery	$ 2383	$ 204	$ 5732	
Indirect costs of death	$ 678	$ 237	$ 3389	Funerary expenses in Colombia, Protection Businees Group [25]

#### Cost-effectiveness criteria

In order to establish whether an intervention is cost-effective, the cost-effectiveness ratio observed must be compared with a cost-effectiveness threshold. Given that the economic evaluation considered two scenarios with different times and outcomes, two different thresholds were considered as well: (1) for a correctly diagnosed CCHD case (1 week of life), a threshold of USD 26.292 was selected (which corresponds to the average direct medical costs of a patient with CCHD); and (2) for probability of survival (at 1 year), a threshold of USD 6.408, the gross domestic product (GDP) per capita in Colombia according to the World Bank [26].

#### Sensitivity analysis to assess the role of uncertainty

Two types of analysis were conducted: (a) a deterministic analysis, which considers point estimates and confidence intervals of each parameter and is presented through a tornado diagram; and (b) probabilistic sensitivity analysis (PSA). In PSA, uncertainty was assessed through Montecarlo simulation and a hypothetical cohort of patients (1000 iterations). A triangular distribution was assigned to costs, and a beta distribution to probabilities and utilities. Results of PSA were illustrated as cost-effectiveness acceptability curves, which show the probability that an alternative is cost-effective for different willingness-to-pay thresholds.

### Budget impact analysis (BIA)

The BIA makes it possible to estimate how much a health system must invest or how much is saved due to the routine use of some technology. The calculation is based on the consideration of two scenarios: a current one, which refers to the treatment indicated for the health condition, with the technologies available within the coverage of the benefits plan for the social security system (SGSSS) or which are being financed with public resources; and a second scenario, called the new one, which describes the treatment that incorporates the new technology or technologies subjected to assessment. The budget impact analysis followed recommendations of the International Society for Pharmacoeconomics and Outcomes Research (ISPOR) [27] and the Colombian Agency of Health Technology Assessment (IETS from its Spanish initials) [28]. Table 4 shows the sources of information used in the calculation of budget impact of a gradual increase in the detection of CCHDs by means of pulse oximetry in the clinical practice in Colombia (Table 4).

**Table 4:** Methodological case of the budget impact analysis

Technologies assessed	(a) General examination with pulse oximetry (b) General examination
Population	Term newborns in Colombia Demographical growth as from DANE projections Epidemiological information (prevalence of CCHDs) taken from SLR
Perspective	Health system—third payer
Time horizon	Every scenario: 1 year Three scenarios are compared
Costs included	See Table 3 (costs associated to the detection of CCHD) The base case corresponds to the current scenario, without the use of pulse oximetry
Sources of information	Costs deriving from the economic assessment Prevalence derived from SLR
Scenarios	Current scenario: pulse oximetry: 0% New scenario a: pulse oximetry 10% New scenario b: pulse oximetry 20%
Results	The difference among estimated scenarios (current and new), expressed as follows: *Budget impact *=* New scenario*–*current scenario*

*DANE* the national administrative department of statistics, *SLR* systematic literature review

Through this analysis, if the result is positive, it is interpreted as the financial effort the country should make to finance this technology. Conversely, if the impact is negative, it means that the country would be saving this cost by using the technology.

## Results

### Economic assessment

For the time horizon of 1 week (outcome of correctly detected cases), the cost of pulse oximetry screening plus the general examination, versus the general examination alone was $102 and $95 respectively. The effectiveness of pulse oximetry plus the general examination, versus the general examination alone was 0.93 and 0.86 respectively. The incremental cost-effectiveness ratio (ICER) was $100 for pulse oximetry screening; that is, if one wishes to increase in 1% the probability of a correct CCHD diagnosis, this amount would have to be invested (Table 5). Under the agreed willingness to pay, pulse oximetry would be cost-effective.

**Table 5:** Cost-effectiveness results baseline case as per outcome

Alternative		Cost	incremental cost	Effectiveness correctly detected case	Incremental effectiveness	ICER correctly detected case
One week	General examination	$95		0.86		
	Pulse oximetry plus general examination	$102	$7	0.93	0.07	$100

Alternative		Cost	Incremental cost	Over life effectiveness	Incremental effectiveness	Survival ICER
First year of life	General examination	$326		0.9745		
	Pulse oximetry plus general examination	$365	$39	0.9755	0.001	$39,050

*ICER* incremental cost-effectiveness ratio

For the results at 1 year of life, considering the mortality associated with CCHD and with medical intervention, the oximetry strategy plus general examination is more effective but more expensive: it would be necessary to invest a large amount of money ($ 39,050) to obtain a 1% increase in survival, when compared with only general physical examination (Table 5).

### Deterministic sensitivity analysis

**Fig. 2:**
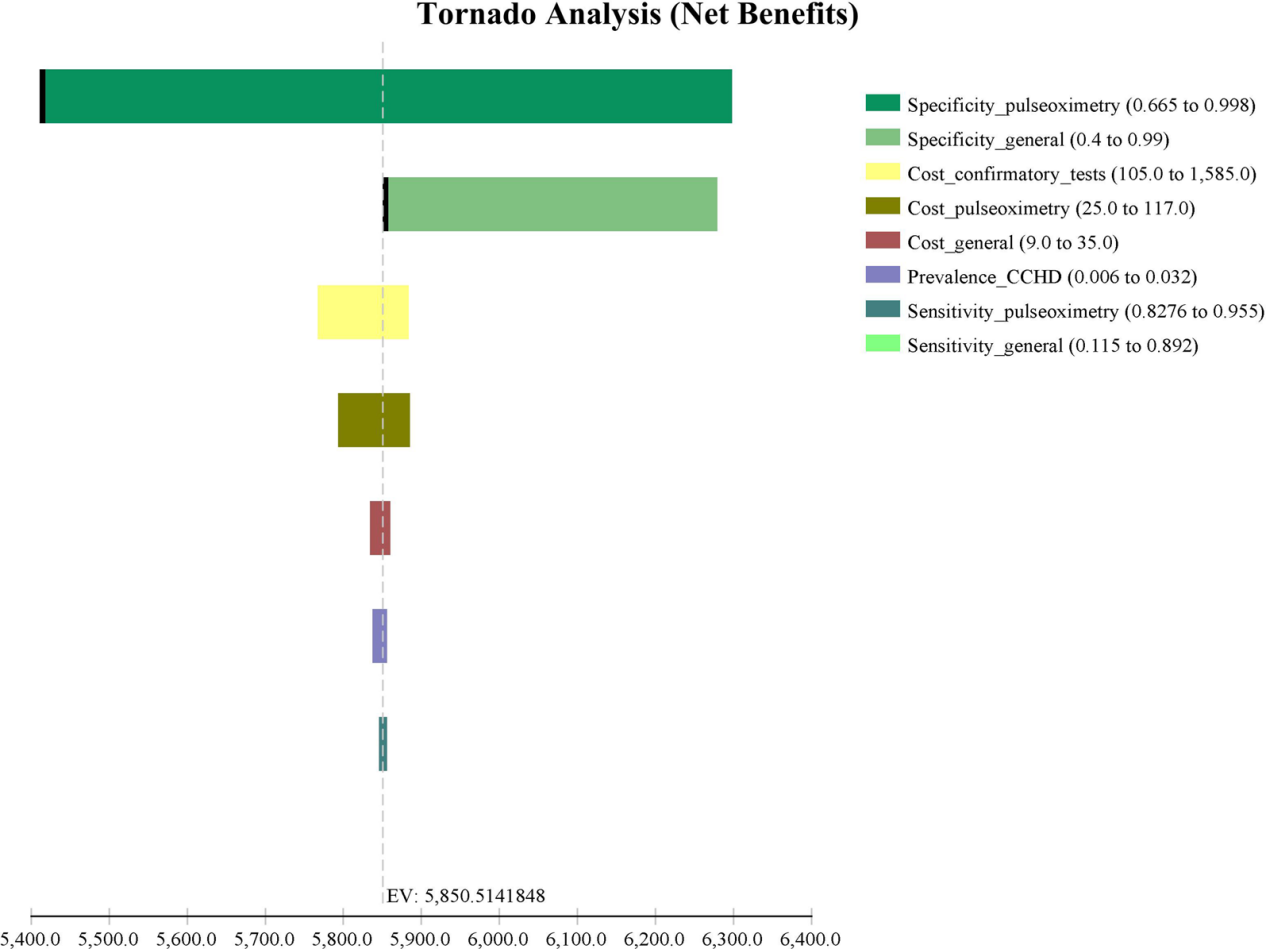
Tornado analysis of results—first week of life

Correctly detected cases: The tornado diagram is shown, and in descending order the variables having most influence on the incremental results (Fig. 2). The most sensitive variables are the specificity of pulse oximetry, followed by costs.

The cost of doing pulse oximetry may be subject to controversy; that is why a one-way sensitivity analysis was performed for this variable. It was found that even with the highest cost of the alternative assessed ($81) the result continues to be cost-effective (Table 6).

**Table 6:** One-way sensitivity analysis: the cost of pulse oximetry

Cost of pulse oximetry	Alternative	Cost	Effectiveness	Incremental cost	Incremental effectiveness	ICER
$ 16	Pulse oximetry plus general examination	$ 77	0.931862	$ –	0	$ –
	General examination	$ 95	0.859246	$ 18	-0.07262	($ 244)
$ 81	General examination	$ 95	0.859246	$ –	0	$ 0
	Pulse oximetry plus general examination	$ 142	0.931862	$ 47	0.072616	$ 653

*ICER* incremental cost-effectiveness ratio

**Fig. 3:**
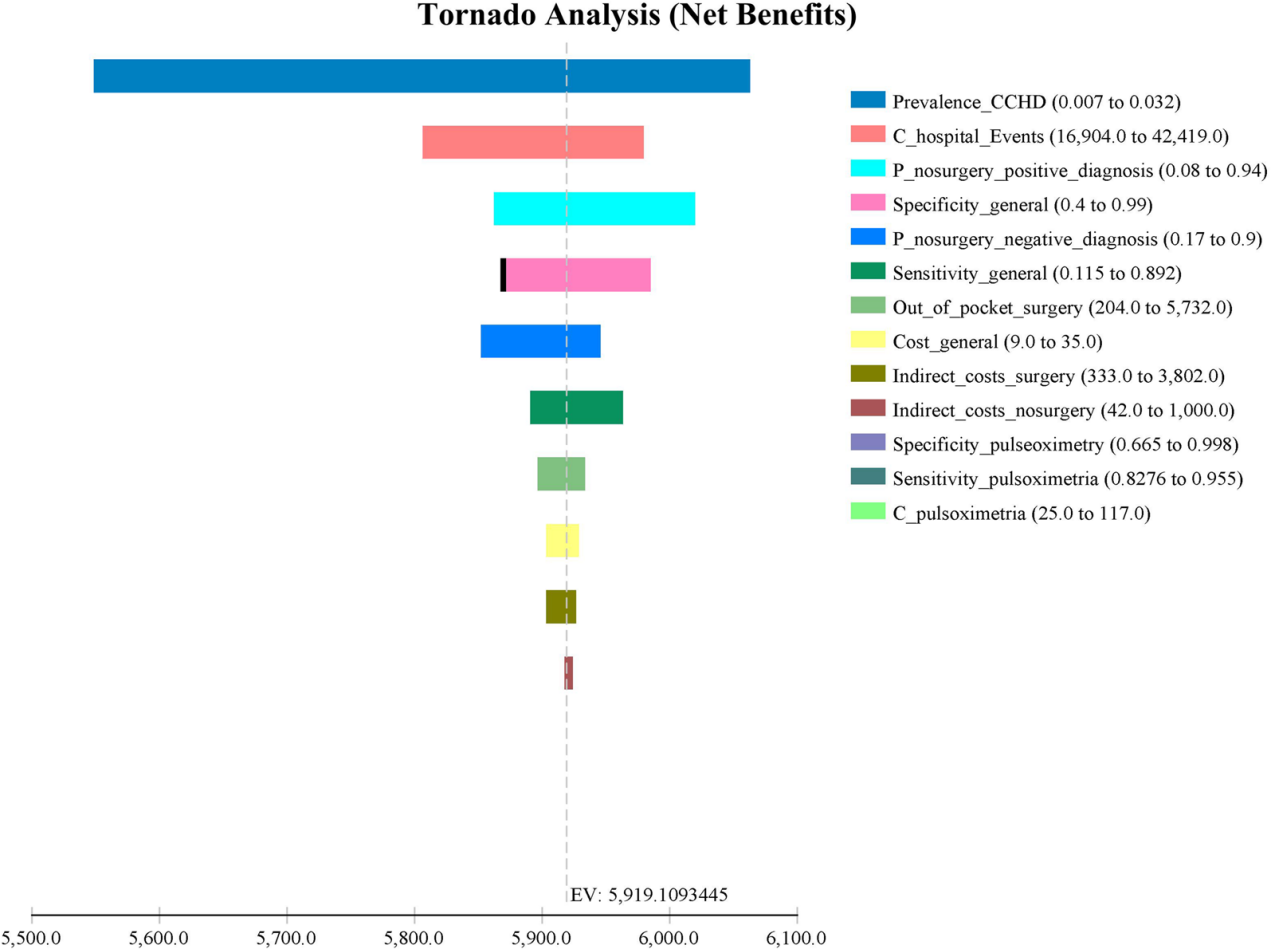
Tornado analysis of results till the first year of life

The tornado analysis of the model up to the first year of life (Fig. 3), the prevalence of CCHD, the probability of surgery among the correctly diagnosed cases, the hospital costs and the specificity of the general physical examination are the variables most influencing the results.

As the prevalence of CCHDs is an element of high impact for the analysis, it was made by taking into account all the possible ranges, whereby for all cases it is necessary to make a high investment ($37,494 to $44,273) to improve effectiveness by 1% as compared with the general examination only (Table 7).

**Table 7:** Sensitivity analysis of one parameter: prevalence

Estimates	Name of the variable	Strategies	Costs	Effectiveness	Incremental cost	Incremental effectiveness	ICER
0.007	Prevalence of CCHD	General physical examination	$ 209	0.978769	$ –	0	$ –
	Prevalence of CCHD	Pulse oximetry + general physical examination	$ 230	0.979258	$ 22	0.000489	$ 44,273
0.032	Prevalence of CCHD	General physical examination	$ 627	0.963658	$ –	0	$ –
	Prevalence of CCHD	Pulse oximetry + general physical examination	$ 711	0.965893	$ 84	0.002235	$ 37,494

*ICER* incremental cost-effectiveness ratio

### Probabilistic sensitivity analysis

**Fig. 4:**
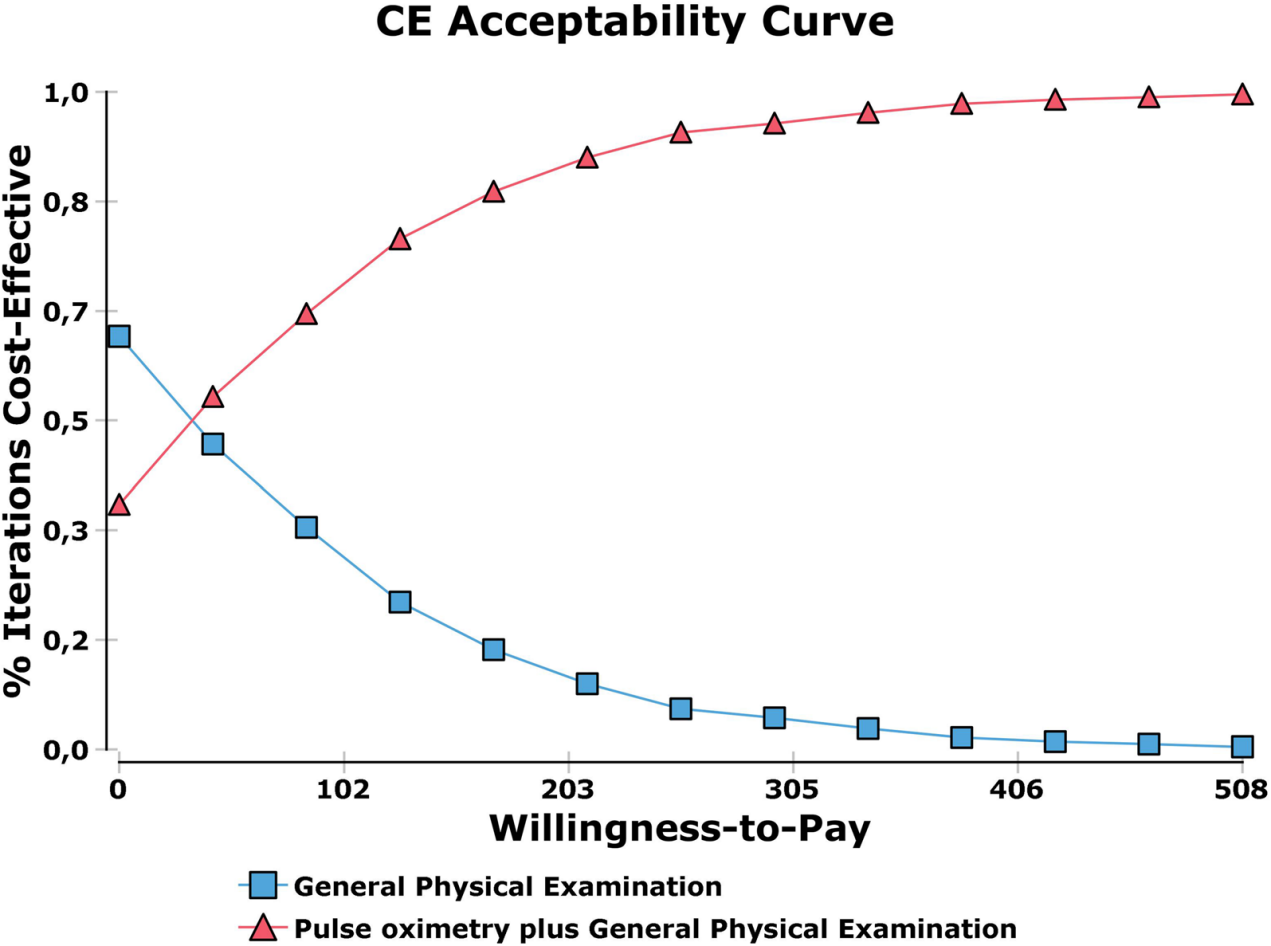
Probabilistic sensitivity analysis: cost-effectiveness acceptability curve

Pulse-oximetry was found to have a larger probability to be cost-effective as the availability to pay increases (Fig. 4).

### Results of budget impact

In the current scenario, a 0% use of pulse oximetry was assumed; thus, the number of cases of CCHD detected are the result of the general physical exam only (2790 cases). For the second year, with the implementation of pulse oximetry in 10% of the newborns, the number of cases detected would be 3241; 425 more cases than in the scenario with screening by the general physical examination only. In the third year, with the implementation for 20% of the cases, 858 cases more would be detected. Table 8 shows the results of the budget impact analysis of the diagnosis and treatment of diagnosed cases. With the scenario of a 10% use of pulse oximetry, the budget impact for the SGSSS is $2,512,359 in the diagnostic phase, and in the assistance of new cases detected, there is an increase of $7,410,700 in costs. These values consider direct medical costs only.

**Table 8:** Budget impact of diagnosis and treatment

	Current scenario	Second year new scenario (10%)	Third year new Scenario (20%)
Detection costs			
Cost of pulse oximetry	$ –	$ 3,357,679	$ 6,821,241
Cost of general physical examination	$ 11,403,687	$ 10,558,368	$ 9,651,464
Total cost of CCHD detection	$ 11,403,687	$ 13,916,046	$ 16,472,705
Budget impact of diagnosis		$ 2,512,359	$ 5,069,018
Costs incurred by detection			
Medical indirect costs	$ 45,911,060	$ 53,321,760	$ 60,862,163
Budget impact of treatment	$ –	$ 7,410,700	$ 14,951,103

*CCHD* critical congenital heart disease

## Discussion

This economic assessment shows that the addition of pulse oximetry to the general physical examination of the newborn, is a cost-effective alternative to correctly detect CCHD cases at birth, with a time horizon of 1 week.. However, using a wider time horizon and considering survival, the strategy would not be cost-effective in Colombia, as it would exceed the cost-effectiveness threshold.

This is the first full economic assessment published on the subject in Latin America, considering the perspective of society and measuring the budget impact for the SGSSS. The results of this study may be compared with other economic assessments around the world. Studies like the one conducted by Peterson et al. in the United States [13] found a cost-effectiveness ratio of USD 40,385 (prices for 2011) per year of life earned, when identifying 1189 additional newborns with CCHD in the hospitals where they were born through pulse oximetry, and preventing 20 additional infant deaths per year. In the United Kingdom, Roberts et al. [14] performed a cost-effectiveness analysis with the same purpose, comparing pulse oximetry as a complement for the clinical examination versus the clinical examination on its own to detect congenital heart disease in newborns. The ICER was £24,000 per case diagnosed in time; they concluded that pulse oximetry is a cost-effective strategy in the light of the threshold defined by the United Kingdom. In China, this strategy was also assessed in the detection of CCHD; the study shows how pulse oximetry reduces the burden of the disease in terms of years of life lost by premature death [15].

The results of the costs estimates showed a high economic impact of CCHD on the Colombian health system and on families. Regarding indirect costs, no studies were found tackling this topic in CCHDs; however, an approximation was made by Raj et al. [29] in a study on patients with congenital heart disease, in which they found that the mean loss of days by parents was 35 and the loss of working days was 15 days on average. Mughal et al. [30.] identified that 12.3% of families contributed totally to the cost associated with the treatment of patients, and 63.1% of families partly contributed to the total cost.

The budget impact with a scenario of 10% was $2,512,359 and for a 20% scenario it corresponds to $5,069,018. Although pulse oximetry is included in the compulsory health plan (POS from its Spanish initials) of the SGSSS, namely the aspects or activities covered by the health system and paid by insurance companies, which are recommended in favor of the correct and timely detection of congenital anomalies, we define this level of percentage implementation because there are still many challenges to overcome before pulse oximetry is taken to clinical practice; among these challenges we find the training of health professionals, the codification of health plans, the forms of contracting with hospitals and the availability of technology in the country, considering that the majority of the population are located in rural areas and rural disperse areas, whereas qualified assistance centers are located in the main cities of the country.

As strengths for this study, none of the studies published in other countries considers a perspective of society nor the budget impact on the country derived from the implementation of the technology assessed. To include the social perspective in the assessment, it was necessary to perform a costs estimate by means of an extensive process which included different sources of information to perform cost estimates considering the costs incurred by the health system and the families. This aspect of the out-of-pocket expenses by the families required an additional effort in order to collect information from primary sources through surveys, considering as well the low prevalence of CCHDs. In addition to this, this study developed a rigorous, reproducible methodology to establish or document probabilities from the opinion of clinical experts.

## Conclusions

From the perspective of the Colombian society and considering a time horizon of 1 week, this economic evaluation shows that a screening strategy of pulse oximetry plus general physical examination is cost-effective in the detection of CCHD in term newborns, when compared with general physical examination alone, This study also provides the necessary information to consider its national implementation for routine use in clinical practice.

## Abbreviations

CCHD: critical congenital heart disease
SGSSS: Colombian General System of Health Social Security
SLR: systematic literature review
WTP: willingness to pay

## Availability of data and materials

The datasets generated and/or analysed during the current study are available in the following link https://www.dropbox.com/sh/lk7mxop3px9zgiy/AABAvbBlHvHY9C2eLO0afmXwa?dl=0
